# Depolarization and CaM Kinase IV Modulate NMDA Receptor Splicing through Two Essential RNA Elements

**DOI:** 10.1371/journal.pbio.0050040

**Published:** 2007-02-06

**Authors:** Ji-Ann Lee, Yi Xing, David Nguyen, Jiuyong Xie, Christopher J Lee, Douglas L Black

**Affiliations:** 1 Department of Microbiology, Immunology, and Molecular Genetics, University of California, Los Angeles, Los Angeles, California, United States of America; 2 Department of Chemistry and Biochemistry, University of California, Los Angeles, Los Angeles, California, United States of America; 3 Molecular Biology Institute, Center for Genomics and Proteomics, University of California, Los Angeles, Los Angeles, California, United States of America; 4 Department of Physiology, University of Manitoba, Winnipeg, Manitoba, Canada; 5 Howard Hughes Medical Institute, University of California, Los Angeles, Los Angeles, California, United States of America; University of Wisconsin, United States of America

## Abstract

Alternative splicing controls the activity of many proteins important for neuronal excitation, but the signal-transduction pathways that affect spliced isoform expression are not well understood. One particularly interesting system of alternative splicing is exon 21 (E21) of the NMDA receptor 1 (NMDAR1 E21), which controls the trafficking of NMDA receptors to the plasma membrane and is repressed by Ca^++^/calmodulin-dependent protein kinase (CaMK) IV signaling. Here, we characterize the splicing of NMDAR1 E21. We find that E21 splicing is reversibly repressed by neuronal depolarization, and we identify two RNA elements within the exon that function together to mediate the inducible repression. One of these exonic elements is similar to an intronic CaMK IV–responsive RNA element (CaRRE) originally identified in the 3′ splice site of the BK channel STREX exon, but not previously observed within an exon. The other element is a new RNA motif. Introduction of either of these two motifs, called CaRRE type 1 and CaRRE type 2, into a heterologous constitutive exon can confer CaMK IV–dependent repression on the new exon. Thus, either exonic CaRRE can be sufficient for CaMK IV–induced repression. Single nucleotide scanning mutagenesis defined consensus sequences for these two CaRRE motifs. A genome-wide motif search and subsequent RT-PCR validation identified a group of depolarization-regulated alternative exons carrying CaRRE consensus sequences. Many of these exons are likely to alter neuronal function. Thus, these two RNA elements define a group of co-regulated splicing events that respond to a common stimulus in neurons to alter their activity.

## Abstract 2

Alternative splicing of NMDA receptor 1 exon 21 is reversibly repressed by depolarization in a CaMK IV-dependent manner in neurons. This suggests splicing is finely tuned by dynamic activity inputs.

## Author Summary

Multiple mechanisms direct changes in neuronal activity in response to external stimuli, ranging from short-acting modifications of membrane proteins to longer-acting changes in gene expression. A frequently regulated step in gene expression is the pre-mRNA splicing reaction in which the inclusion of exons (protein-coding sequences) or the position of splice sites produces alternatively spliced mRNA isoforms encoding functionally different proteins. Here, we study splicing of the NMDA receptor, which responds to the neurotransmitter glutamate to modify neuronal activity. We show that the splicing of an important exon (E21) in the NMDA receptor subunit NR1 mRNA is repressed by cell depolarization and activation of the intracellular signaling molecule, CaMK IV. We find that this splicing repression is mediated by two regulatory sequences within the exon itself. One sequence is similar to a previously described regulatory element that had not been known to function in an exon. The other is a new element. The characterization of these elements as a family of degenerate sequences allowed the identification of a group of exons sharing responsiveness to cell depolarization and CamK IV. These results define a new set of gene expression changes that may occur in modulating neuronal activity.

## Introduction

N-methyl-D-aspartic acid (NMDA)-sensitive glutamate receptors (NMDARs) play important roles in modulating synaptic function in the brain [1,2]. Functional NMDARs are heteromeric complexes assembled from one NR1 subunit and one or more NR2 subunits (NR2A, NR2B, NR2C, and NR2D). The NR1 subunit is encoded by a single gene with three alternatively spliced exons that can generate eight different variants. Exon 5 encodes an optional portion of the extracellular N-terminal domain that regulates the pharmacological properties of the receptor. Alternative 3′ splice sites in exon 22 determine the choice of variant intracellular C-terminal domains that affect the rate of export from the endoplasmic reticulum (ER). Regulated exon 21 encodes a peptide cassette that modulates a variety of activities of the protein, including trafficking from the ER to the plasma membrane, phosphorylation by protein kinase C (PKC) and protein kinase A (PKA), interactions with yotiao, neurofilament L, and calmodulin, and activation of NMDAR-induced gene expression [3–7]. The splicing of these alternative exons is regulated at different developmental stages and locations, and can be altered by neuronal activity [8–10]. Hence, alternative splicing has a profound effect on the function of NMDARs.

The spliceosome is a large complex of small nuclear ribonucleoprotein particles (snRNPs) that assemble onto splice sites to bring the intron ends into juxtaposition, and catalyze intron excision and exon ligation [11]. Alternative splicing results from changes in the choice of splice sites by the spliceosome, and is mediated by numerous RNA elements in the pre-mRNA [12,13]. These control elements can be either exonic or intronic, and can act as either enhancers or silencers. Individual elements are bound by specific regulatory factors that include members of the serine/arginine-rich (SR) family of proteins [14,15] and the heterogeneous nuclear ribonucleoprotein (hnRNP) group of proteins [16]. The SR proteins generally enhance splicing via recognition of exonic splicing enhancers (ESEs), although they can act in other ways. The hnRNP factors can play diverse roles in enhancement and repression. Some splicing factors are variably expressed in different cell types to generate tissue-specific splicing patterns. Splicing patterns can also respond to stimuli within a particular cell [17,18], yet how this dynamic regulation of splicing occurs is poorly understood.

Given its importance to neuronal function, NR1 exon 21 (E21) is a particularly interesting model to study splicing regulation. E21 is included prominently in the forebrain and more frequently skipped in the hindbrain [19]. Several SR proteins as well as the RNA binding proteins NAPOR/CUGBP2, hnRNP H, and NOVA2 are thought to positively regulate this exon [19–21]. Identified splicing enhancer sequences for E21 include binding sites of the SR proteins ASF/SF2, SC35, and SRp40 within the exon, and binding sites for hnRNP H and NAPOR/CUGBP2 in the downstream intron. In contrast, hnRNP A1 can repress the splicing of this exon via its interaction with two exonic UAGG motifs and one intronic G tract [20]. The interactions between these many different proteins and RNA motifs are thought to contribute to the complex patterns of splicing seen for E21 within the brain.

In neurons, several exons are known to undergo changes in splicing when the cells are shifted to depolarizing media [10,22]. We previously showed that the repression of the BK channel STREX exon after depolarization requires calcium ion influx through L-type calcium channels and a downstream Ca^++^/calmodulin-dependent protein kinase (CaMK) [23]. We found that STREX, as well as the NR1 exons 5 and 21, were repressed by overexpressed CaMK IV. A CaMK IV–responsive RNA element (CaRRE) was identified in the 3′ splice site of the STREX exon [24]. In searches for this element, the 3′ splice sites of NR1 exon 5 and several other exons were found to contain a CaRRE and be subject to repression by CaMK IV. However, the CaRRE was not sufficiently defined to identify many additional CaMK-responsive exons. It was not clear how widespread CaRREs were in the genome, whether there might be additional types of calcium-responsive elements, or whether these elements defined sets of coregulated exons. Moreover, the NR1 E21 3′ splice site did not contain a CaRRE motif. The source of the responsiveness of this exon remained unclear, and we wanted to understand how it could respond to stimuli.

In this study, we characterize the response of NMDAR1 E21 to depolarization and activated CaMK IV. We identify two RNA elements within E21 that determine its dynamic regulation. We identify a group of additional exons containing these elements that can also be regulated by depolarization, and define a set of splicing events that likely play important roles in modulating the neuronal activity.

## Results

### Depolarization Induces NR1 E21 Repression in Differentiated P19 Cells

**Figure 1:**
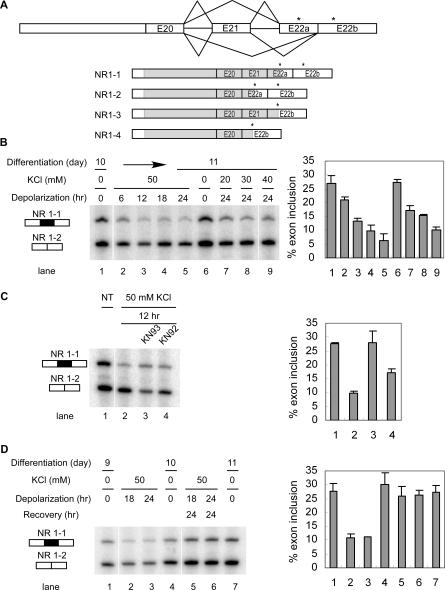
Depolarization Induces Splicing Repression of NMDAR1 Exon 21 in Differentiated P19 Cells (A) Top: diagram of the alternative splicing at the 3′ end of NMDAR1 pre-mRNA. Exon 21 is an alternative exon that can be spliced to either exon 22a or 22b through the use of alternative 3′ splice sites. Asterisks (*) indicate the stop codons in E22a and E22b. Bottom: the four isoforms of the spliced mRNA. The protein-coding region is depicted as a gray bar in the mRNA. (B) Depolarization represses the splicing of E21 to E22a in differentiated P19 cells. Left panel: denaturing gel electrophoresis of the NR1–1 and NR1–2 RT-PCR products from day 10 differentiated P19 cells depolarized with 50 mM KCl for 0, 6, 12, 18, or 24 h (lanes 1–5), from day 11 untreated cells (lane 6), and from day 10 cells depolarized with 20, 30, or 40 mM KCl for 24 h. Right panel: percentage of mRNA containing E21 after depolarization ((NR1–1/(NR1–1 + NR1–2))*100). Bars indicate mean ± standard deviation (s.d.), *n* = 3. (C) Depolarization-induced repression is dependent on CaMK. Left panel: RT-PCR assay of NR1–1 and NR1–2 mRNA from differentiated P19 cells treated with KCl, KCl plus KN93, or KCl plus KN92 for 12 h. Right panel: graph of E21 inclusion as shown in the left panel. (D) Splicing repression is reversible after removal of the stimulus. Left: RT-PCR assay of NR1–1 and NR1–2 mRNA isolated from cells depolarized with 50 mM KCl for 18 or 24 h and 24 hours after the salt washout. Right: graph of E21 inclusion as shown in the left panel.

Previously, we showed that splicing of NMDAR1 E21 is repressed by CaMK IV after transient expression of a minigene reporter in HEK 293T cells. [23]. It was not clear whether E21 in endogenous NR1 transcripts could be similarly repressed by depolarization and CaMK IV. To confirm E21 repression in endogenous NR1 transcripts, we examined its splicing in differentiated P19 embryonal carcinoma (EC) cells by RT-PCR. Undifferentiated cells were aggregated in retinoic acid (RA) and then dispersed and replated [25]. Ten days after RA treatment, 60% of the cells exhibited neuronal morphology with extended fasciculated processes synapsing on other cells. The remainder of the culture consisted of flat adherent cells that resembled either astrocytes or undifferentiated EC cells [26]. The alternative splicing of E21 to E22a or E22b at the 3′ end of NMDAR1 transcript results in four possible different NMDAR1 isoforms. We used E22a- or E22b-specific reverse primers to separately measure the NR1–1:NR1–2 and NR1–3:NR1–4 ratios (Figure 1A). All four NR1 mRNA isoforms were detected in differentiated P19 cells, with 26% of E21 inclusion in the NR1–1/NR1–2 isoform pair (Exon 22a; Figure 1B, lane 1) and 12% inclusion in the NR1–3/NR1–4 isoform pair (Exon 22b; Figure S1A). To examine the effect of depolarization on splicing, these cells were exposed to 50 mM KCl. E21 inclusion was reduced after 6 h of this treatment, and continued to decrease to about 25% of its original level after 24 h (Figure 1B, lanes 2–5, and Figure S1A). The extent of repression was dependent on the KCl concentration; in 30 mM KCl, E21 splicing was reduced by 50% at 24 h, whereas in 50 mM KCl, E21 splicing was reduced by 75% (Figure 1B, lanes 5 and 8). These E21 changes were observed in both exon 22a– and exon 22b–containing mRNA. Thus, E21 splicing is indeed repressed by depolarization, and this repression is apparently independent of the choice of alternative 3′ splice site downstream.

We next tested whether the CaMK pathway is involved in the depolarization-induced repression, as seen with the BK channel STREX exon. Treatment of the cells with the CaMK inhibitor KN93 prior to depolarization completely blocked the splicing repression (Figure 1C, lane 3, and Figure S1B). The less-active analog KN92 showed an intermediate effect (Figure 1C, lane 4). Thus, depolarization-induced splicing repression of E21 is mediated through the CaMK pathway in differentiated P19 cells, similar to what was observed for BK channel splicing in the pituitary cell line GH_3_.

The observation of a splicing change after a stimulus requires removal of the pre-existing mRNA. Thus, the shift in splicing is observed in a timeframe of hours in response to a continuous application of high KCl concentrations. Although we did not observe a decrease in cell viability from the treatment, this splicing alteration could derive from a permanent change in the cells after chronic depolarization. Alternatively, the E21 skipping could be a reversible adaptation to the depolarizing media. To test the reversibility of this splicing repression, KCl was washed out after 18 or 24 h of depolarization, and then the cells were grown for another 24 h. Removal of the high concentration of KCl from the media restored E21 splicing to its normal level (Figure 1D, lanes 5 and 6, and Figure S1C). Thus, the splicing repression is reversible and is a dynamic response to environmental cues.

### The Exonic Sequence of E21 Is Sufficient for CaMK IV–Induced Splicing Repression

**Figure 2:**
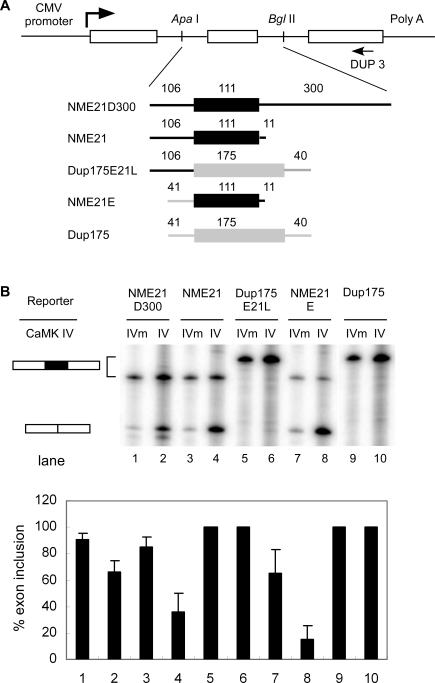
The Exonic Sequence of NR1 E21 Is Sufficient for CaMK IV–Induced Splicing Repression in HEK 293T Cells (A) Top: diagram of the pDup minigene reporter. The reporter has a three-exon structure, and the second exon can be replaced with other exons between ApaI and BglII sites in the introns. The CMV promoter, exons (boxes), introns (lines), and primer extension primer DUP 3 (arrow) are indicated. Bottom: structures of hybrid second exons. Sequences derived from human NR1 E21 are depicted as black lines and boxes; sequences derived from *β*-globin sequences of the second exon in Dup175 construct are depicted as grey lines and boxes. The lengths of these exons and intron segments are indicated above the exon diagram, and names of these reporters are indicated to the left of the exon diagram. (B) Top: primer extension assay of reporters co-expressed with CaMK IV-dCTK75E (IVm), or CaMK IV-dCT (IV). Bottom: graph of exon inclusion from the gel above.

To study the sequence elements needed for E21 regulation, we cloned the human E21 exon with flanking intron sequence between two constitutive *β*-globin exons of the Dup4–1 plasmid (Figure 2A). This reporter was transiently expressed in HEK 293T cells, and the mRNAs were assayed for E21 inclusion by primer extension. The repression of E21 by CaMK IV was examined by co-transfection of plasmids expressing a constitutively active form of CaMK IV (CaMK IV dCT) or a kinase-dead mutant control (CaMK IV dCT K75E). E21 was strongly included in the NME21D300 mRNA (91%) when co-expressed with the dead CaMK IV (dCT K75E). In contrast, E21 splicing was reduced by the active CaMK IV (67% inclusion; Figure 2B, lanes 1 and 2).

To roughly localize the RNA elements responsible for CaMK IV repression, we made several chimeric minigenes containing portions of E21 and portions of the constitutive Dup175 exon, and examined their response to CaMK IV. Deletion of the downstream intron (NME21) had little effect on the overall splicing, but made E21 more repressible by CaMK IV dCT (Figure 2B, lanes 3 and 4). The deleted downstream intron sequence presumably contains positive elements that counteract the repression. As seen previously, and unlike the BK channel STREX exon or NR1 exon 5, the E21 3′ splice site does not confer CaMK IV responsiveness when transferred to the constitutive Dup175 exon (Dup175E21L; Figure 2B, lanes 5 and 6). In contrast, the E21 exon alone, when fused to the Dup175 exon 3′ splice site (NME21E), was highly repressed by CaMK IV (Figure 2B, lanes 7 and 8). These results indicate that the E21 exonic sequence is sufficient to induce CaMK IV–dependent splicing repression. This is in contrast to other identified exons in which the regulatory element is located in the 3′ splice site.

### A CaRRE-Like Element and a Purine-Rich Sequence Are Both Needed for Efficient CaMK IV Repression of E21

**Figure 3:**
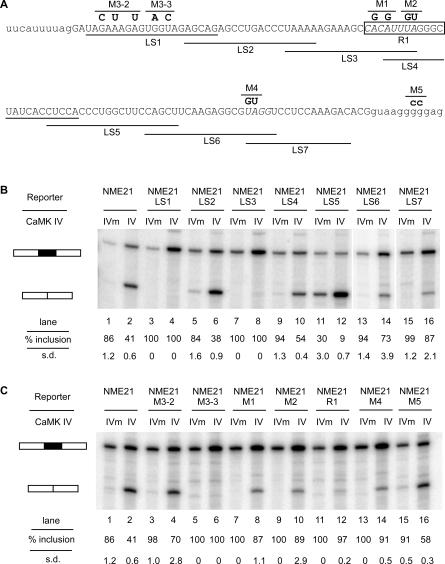
Exon Scanning and Point Mutagenesis Reveal Sequences Needed for CaMK IV–Induced Splicing Repression (A) The exonic sequence and splice sites of human E21. The replacement of 20 nucleotides of exon sequence with the IgM sequence UCAGCAUGACUCUCAGCAUG is indicated by a line under the replaced sequence, and the clone name is below the line. LS7 carried only a 16-nucleotide exon replacement with the first 16 nucleotides of the IgM sequence. Specific mutations are indicated by bold type above the nucleotides, and the clone name is above the mutation. The R1 mutation is generated by replacing the boxed sequence with the IgM sequence CAUGACUCUCAG. The predicted sites of the CaRRE 1 and the hnRNP A1 binding site are italicized. (B) Primer extension assay of the LS mutants co-expressed with CaMK IV-dCTK75E (IVm) or CaMK IV-dCT (IV). Percentages of exon inclusion and standard deviations (*n* = 3) are indicated beneath the lanes. (C) Primer extension assay of specific point mutants co-expressed with CaMK IV-dCTK75E (IVm) or CaMK IV-dCT (IV). Percentages of exon inclusion and standard deviations (*n* = 3) are indicated beneath the lanes.

To identify the sequence elements involved in E21 repression, we performed linker scanning mutagenesis across the exon. Blocks of 20 nucleotides within the E21 exon were sequentially substituted with a 20-nucleotide sequence from the IgM gene that is known to lack splicing activity [27]. To maintain the 3′ and 5′ splice sites, the sequence substitutions started at exon nucleotide 3 and ended four nucleotides from the 3′ end of the exon (Figure 3A).

Two different substitution mutations had strong effects on the CaMK IV–induced repression. The response was completely eliminated when nucleotides 3–22 (LS1) or nucleotides 33–52 (LS3) were substituted with the heterologous sequence (Figure 3B, lanes 3 and 4 [LS1], and 7 and 8 [LS3]). Thus, at least two RNA elements, one from each region, are needed together for the CaMK IV–induced repression. Having either one of these RNA elements alone is not sufficient for this response. As described earlier, several ESEs and exonic splicing silencers (ESSs) have been identified in the E21 exon, and we found that a substitution that eliminated these ESEs (LS5) did reduce the overall exon inclusion [20,28] (Figure 3B, lanes 11 and 12). However, the CaMK IV response was not affected by this mutation, because the exon was still more excluded in the presence of the activated CaMK IV.

We showed previously that the splicing repression of the BK channel STREX exon is mediated by a CaRRE (CACAUNRUUAU) located in the 3′ splice site. Sequence analysis of the LS3 region identified a CaRRE-like sequence CACAUUUA within it. Point mutations (M1 and M2) within this CaRRE-like sequence led to significant decreases in CaMK IV repression (Figure 3C, lanes 7–10). A larger 12-nucleotide substitution of the entire CaRRE motif (R1) nearly eliminated the CaMK IV effect (Figure 3C, lanes 11 and 12). Interestingly, the LS4 scanning mutation also changes some nucleotides in this region, but only gives a small reduction in splicing repression. Subsequent analysis of this element indicates that by chance, the LS4 mutations only have a small effect on activity (see below). These results identify a functional CaRRE motif within the LS3 region and indicate that this motif can function within an exon as well as the 3′ splice site.

In contrast to LS3, no known ESS was identified in the LS1 region. To map the important elements within this sequence, we mutated a purine-rich element (M3–2) and the adjacent sequence downstream (M3–3; Figure 3A). Although the mutation M3–2 had no effect on CaMK IV repression, the mutation M3–3 totally abolished this response (Figure 3C, lanes 5 and 6), identifying critical nucleotides in the LS1 region. We called this sequence a CaRRE type 2 motif and will refer to the previous CaRRE as a CaRRE type 1 motif.

Recently, exonic UAGG and intronic G track motifs were shown to be important in silencing E21 splicing [20]. The possible roles of these elements in the CaMK IV–dependent repression were examined with mutations M4 and M5. Consistent with the previous observations, mutation of the UAGG motif promoted E21 splicing in our system (Figure 3C, lanes 13 and 14). The M4 mutant also exhibited a significantly weaker CaMK IV response. Thus, the reduced exon repression observed in LS6 and LS7 mutants is presumably due to the loss of this element. In contrast, disruption of the G track had no effect on CaMK IV response. These results indicate that CaMK IV–mediated exon repression requires multiple elements, and that there are two critical elements separate from the UAGG and G track motifs.

### Either CaRRE Is Sufficient for CaMK IV Repression in a Heterologous Exon

**Figure 4:**
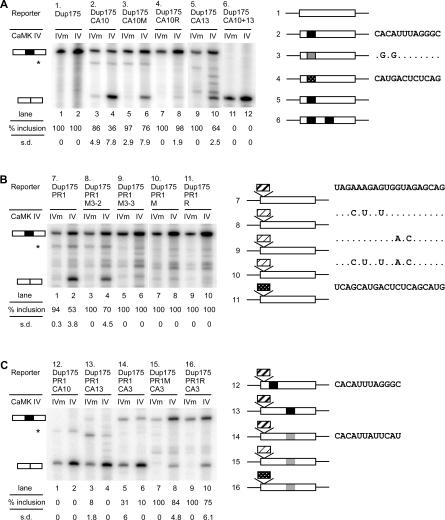
Either Exonic CaRRE Is Sufficient for CaMK IV Repression in a Heterologous Exon (A) Right: diagram of the chimeric exons generated by replacing nucleotides 13–24 or 72–83 of exon 2 in the Dup175 minigene with the NR1 E21 CaRRE 1 sequence (NR1 E21 nucleotides 45–56, filled box), a mutant sequence (gray box), or an unrelated IgM sequence (dotted box). The clone numbers and the replacement sequences are indicated to the left and right, respectively. Left: primer extension assay of the minigenes indicated above. (B) Right: diagrams of the chimeric exons generated by inserting NR1 E21 nucleotides 3–22 (CaRRE 2, dark-hatched box), a mutant sequence (light-hatched box), or an unrelated IgM sequence into Dup175 exon at the +3 position. The clone numbers and the inserted sequences are indicated to the left and right, respectively. Left: primer extension assay of the minigenes indicated above. (C) Right: Dup175PR1CA10 and Dup175PR1CA13 exons are generated by replacing nucleotides 33–44 or 92–103, respectively, in the Dup175PR1 exon with the NR1 E21 CaRRE 1 sequence. Dup175PR1CA3, Dup175PR1MCA3, and Dup175PR1RCA3 were generated by replacing the E21 CaRRE 1 sequence with the NR1 E5 intronic CaRRE 1 sequence (gray box). Left: primer extension assay of the minigenes indicated above. The asterisk (*) indicates a cryptic splicing event.

Although the two exonic elements in E21 are both needed for efficient CaMK IV repression, we wanted to test if they could function independently in other exons. For this, we transferred the CaRRE 1 motif to the constitutive Dup175 exon and characterized its response (Figure 4A). Introduction of CaRRE 1 into the 5′ region of Dup175 exon (Dup175CA10) caused slight exon skipping, indicating that CaRRE 1 is a weak splicing silencer. However, co-expression of this clone with CaMK IV strongly repressed exon inclusion (Figure 4A, lanes 3 and 4). This effect was due to the introduced CaRRE 1, because a small point mutation in the element reduced its effect, and replacement with a neutral IgM sequence at the same position in Dup175 exon was not able to confer the repression (Figure 4A, lanes 5–8). Thus, the CaRRE 1 motif can be sufficient to silence splicing within an exonic context. To investigate the effect of position on the activity of CaRRE 1, the element was moved to the middle of the Dup175 exon (Dup175CA13). This centrally located CaRRE 1 behaved similarly to an element upstream, but gave a somewhat reduced response to CaMK IV (Figure 4A, lanes 9 and 10). When two CaRRE 1 motifs were introduced in the Dup175 exon (Dup175CA10+13), splicing was completely repressed even in the absence of CaMK IV activity (Figure 4A, lanes 11 and 12). Thus, CaRRE 1 is a splicing silencer whose activity is dependent on both the sequence context and the number of elements in the exon.

Similarly, the CaRRE 2 motif was also tested for its ability to function independently. CaRRE 2 was inserted at the +3 position of the Dup175 exon, the same position relative to the 3′ splice site as in E21 (Figure 4B). Similar to CaRRE 1, CaRRE 2 had weak splicing silencer activity on its own, slightly reducing exon inclusion of the new exon. When co-expressed with CaMK IV dCT, exon skipping was sharply increased, indicating that CaRRE 2 is also sufficient for CaMK IV–dependent repression (Figure 4B, lanes 1 and 2). To confirm that this effect was due to the same element identified in E21, the same mutations were tested. As before, M3–2 had a weak effect on the inducible repression (Figure 4B, lanes 3 and 4). M3–3, the combined mutation (M), and a replacement sequence (R) all eliminated the repression activity, showing that M3–3 mutation covered the minimal CaRRE 2 sequence (Figure 4B, lanes 5–10).

The interaction between the CaRRE 2 and CaRRE 1 motifs was examined by introducing CaRRE 1 to positions downstream of CaRRE 2 (Figure 4C), mimicking the E21 situation. Similar to two copies of CaRRE 1, one copy of each silencer element led to the complete repression of the exon (Figure 4C, lanes 1–4). To weaken the activity of CaRRE 1, we changed the inserted CaRRE 1 sequence to that of the CaRRE found in the 3′ splice site of NR1 exon 5 (Dup175PR1CA3). This partially restored splicing to 31% inclusion. Overexpression of CaMK IV reduced the splicing to 10% (Figure 4C, lanes 5 and 6). Similarly, for these exons with both CaRRE 1 and CaRRE 2, mutations in CaRRE 2 restored splicing in the absence of CaMK (Figure 4C, lanes 7 and 9). These exons were still repressed by the CaMK IV, and behaved like the exons with a single CaRRE motif (Figure 4C, lanes 8 and 10). These results indicate that the level of constitutive and inducible repression from the CaRRE's can be finely tuned by the number, position, and exact sequence of the elements present in the exon.

### Single Nucleotide Scanning Reveals the Degenerate Nature of CaRREs

Most splicing regulatory elements exhibit considerable degeneracy in the sequence that can bind to a particular regulatory protein and affect splicing. The intronic and the exonic CaRRE 1 have the same CACAY core sequence, but are different in the 3′ half and have different activities when placed at the same position in the Dup175PR1 exon (Figure 4C, Dup175PR1CA13 and Dup175PR1CA3). Moreover, several CaRRE 1 mutants also have moderate activity, suggesting that CaRRE 1 might have other functional variants, but there is little information on which nucleotides are most important to their function. Similarly, very little is known about the sequence requirements for CaRRE 2 activity. The finding that exonic CaRRE motifs can also affect splicing indicates that there are likely to be many more exons regulated by CaRRE 1 or CaRRE 2. Thus, we needed to characterize the CaRRE in more detail before searching for them in other exons.

To characterize the functional sequence of these two CaRREs, we carried out single nucleotide scanning mutagenesis of CaRRE 1 and CaRRE 2. We mutated each CaRRE nucleotide to the other three possible nucleotides, one at a time. To facilitate cloning, we first changed the ApaI site in the Dup construct upstream intron to a XhoI site. Interestingly, this change reduced the CaMK IV responses of both the 175CA10-X and 175PR1-X exons compared to their original sequences. The original ApaI site contains a GGG motif that may weaken the exon and increase the silencing activity of the CaRREs.

**Figure 5:**
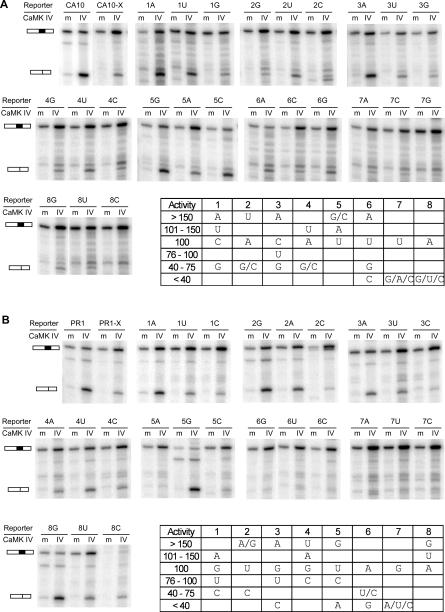
Degenerate Sequences Can Act as CaRRE 1 and CaRRE 2 (A) and (B) Top: single nucleotide scanning mutagenesis of CaRRE 1 and CaRRE 2. The ApaI site of pDup175 was mutated to XhoI site to facilitate the cloning. Each nucleotide of CaRRE 1 (A) and CaRRE 2 (B) was mutated to the other three possible nucleotides, one at a time. Each mutant was co-transfected with CaMK IV-dCTK75E (m) or CaMK IV-dCT (IV) Bottom right of (A) and (B): summary of the relative activity of the CaRRE mutants. The activities of wild-type CaRREs were defined as 100%, and the relative activity of each mutant was calculated by normalizing the difference of the percentage of exon inclusion caused by CaMK IVdCT to the difference observed in its wild-type CaRRE. These mutants are classified by their relative activities (left column). The 8A to 8C mutation in CaRRE 2 disrupted the splicing of this minigene, and the relative exon inclusion could not be determined.

In the absence of CaMK IV, most mutations did not have much effect on the splicing of the exons. When co-transfected with CaMK IV dCT, the different CaRRE mutants exhibited differential responses to the CaMK IV (Figure 5A and 5B). To evaluate these differences, we defined the wild-type silencing activity as 100% and calculated the relative activity of each mutant (Figure 5).

As expected, many mutations reduced the CaMK IV repression to less than 75% of the wild-type activity, and there were four CaRRE 1 mutations and seven CaRRE 2 mutations that only moderately affected silencing activity (between 150% and 75% of their corresponding wild-type activity). Interestingly, there were six CaRRE 1 mutants and six CaRRE 2 mutants that significantly improved silencing activity to greater than 150% of wild type. Among these, a U to G or U to C mutation at position 5 in CaRRE 1 generated a 3-fold increase in silencing activity. In CaRRE 2, a U to G mutation at either position 2 or position 5 also gave a 3-fold increase in the silencing activity. These data indicate that both CaRRE 1 and CaRRE 2 can function through degenerate sequence elements and are likely more abundant than previously expected.

### The Consensus CaRREs Identify a Group of Depolarization-Responsive Exons within the Mouse Genome

**Figure 6:**
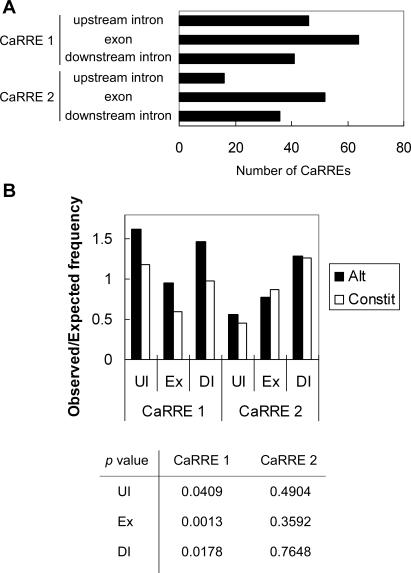
Genome-Wide Identification of Alternative Exons with CaRRE 1 and CaRRE 2 Sequences (A) A database of 2,461 alternatively spliced exons was searched for the family of degenerate CaRRE 1 and CaRRE 2 sequences. The search included the exon and 50 nucleotides of upstream and downstream intron sequences. The number of CaRREs found in these different regions is illustrated. (B) CaRRE motif frequencies. The CaRRE 1 and CaRRE 2 motif frequencies in the upstream intron (UI; position −50 to −1), the exon (Ex), and the downstream intron (DI; position 1 to 50) were calculated separately from a dataset of 2,461 alternative exons (Alt) and a dataset of 9,401 constitutive exons (Constit). The observed CaRRE frequency was calculated by dividing the number of CaRREs by the number of octamers in the searched region. This observed frequency was then normalized to the expected frequency of the random octamers. The *p*-values are given below the histogram.

To identify exons that might be regulated by depolarization, we searched for CaRRE 1 and CaRRE 2 alone and in combination in a dataset of 2,594 mouse alternative exons. Exons are generally between 50 and 250 nucleotides in length, and only exons of length shorter than 253 nucleotides were examined (2,461 exons). Each exon and 50-nucleotide regions of upstream and downstream intron were separately searched for a list of putative CaRRE motifs. The list of CaRRE sequences included all those with an activity of at least 40% of the wild-type elements. The search identified 151 CaRRE 1 motifs and 104 CaRRE 2 motifs within 248 alternative exons or their flanking introns (Figure 6A). Since NR1 E21 did not pass our automatic expressed sequence tag (EST)-genome mapping and filtering for cassette exons (see Materials and Methods), it was not included in the searchable alternative exon set. There were 13 alternative exons that contained two CaRRE motifs. Of these, three alternative exons contain one exonic CaRRE 1 and one exonic CaRRE 2, as seen in NR1 E21.

About 10% of the alternative exons were found to contain potential CaRRE 1 or CaRRE 2 motifs, suggesting that these elements contribute to the regulation of many alternative exons. To evaluate the significance of these CaRRE motifs in alternative exons, we compared the CaRRE frequencies in alternative exons and constitutive exons. We compiled a database of 10,000 constitutive exons, from which 9,401 exons with lengths shorter than 253 nucleotides were used in the calculation. The observed CaRRE motif frequencies in these two exon groups were calculated by dividing the number of CaRREs by the number of octamers in the searched region and then normalizing to the frequency of random octamers. The normalized CaRRE motif frequency distributions in alternative exons and constitutive exons are plotted separately for CaRRE1 and CaRRE 2 in Figure 6B. From these calculations, CaRRE 1 motif frequencies were found to be significantly higher in alternative exons than in constitutive exons, and also significantly higher in the introns flanking alternative exons. In contrast, we did not observe a significant enrichment of CaRRE 2 motifs in these regions encompassing alternative exons relative to constitutive exons. Since this element can clearly affect splicing, this lack of enrichment could be due to the CaRRE 2 sequence not being sufficiently defined.

**Figure 7:**
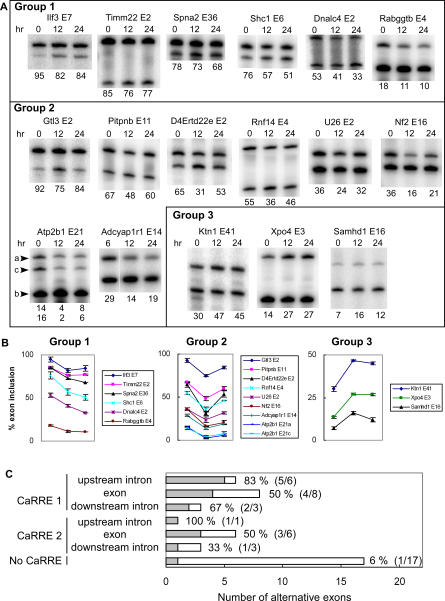
RT-PCR Examination of Depolarization-Induced Splicing Changes of CaRRE-Containing Exons in Differentiated P19 Cells (A) Regulated alternative exons. Sixteen CaRRE-containing exons and one control alternative exon showed splicing changes in the semi-quantitive RT-PCR analysis (22–25 cycles). Each gel panel shows the splicing patterns in rested cells (0 h) and cells treated with 50 mM KCl for 12 and 24 h. These exons are classified by their responses to depolarization. Group 1 contains exons whose splicing was repressed through 24 h. Group 2 contains exons whose splicing was repressed at 12 h, but recovered at 24 h. Group 3 contains exons whose splicing was enhanced by depolarization. The use of alternative 5′ splice sites in Atp2b1 E21 generates two isoforms that are indicated by a and c, and the exon-skipped isoform is indicated by b to the left of the gel. (B) Graphs of exon inclusion as shown above. (C) Summary of the semi-quantitative RT-PCR for the selected alternative exons. The regulated alternative exons are depicted as a gray bar and the non-regulated alternative exons are depicted as an open bar. The percentages of regulated alternative exons among the tested alternative exons in each category are indicated to the right of the bars, and the numbers of regulated and tested alternative exons are shown in the parentheses. More information is summarized in Table 1 and Table S1.

We next tested whether the presence of a CaRRE motif in the exon is predictive of depolarization-induced repression. Sixty exons from different categories were chosen for analysis, and of these, expression of 45 exons was detected by RT-PCR in differentiated P19 cells (unpublished data). Most of these exons showed partial inclusion, confirming their categorization as alternative exons. From this set, 27 exons were selected for further analysis. Because every exon has different splicing efficiency in these cells, we used one of two criteria to categorize the splicing as changed after depolarization. If an exon shows an intermediate level of inclusion between 30% and 70%, it is easier to detect change in splicing. For these exons, a 15% change in inclusion was considered significant. In contrast, when an exon is spliced in less than 30% or greater than 70% of transcripts, it is more difficult to detect a large percent change in splicing. For this group, responsive exons were defined as those showing more than a 2-fold difference in exon inclusion or skipping by depolarization. By these criteria, 13 exons were repressed after depolarization (Figure 7A and 7B), and can be classified into two groups by their responses to the depolarization. Group 1 contained six exons whose splicing remained repressed through 24 h, as was seen for NR1 E21. Group 2 contained seven exons whose splicing was repressed at 12 h, but then began to recover after 24 h under depolarizing conditions. These exons apparently adapt to the condition of chronic depolarization and are initially repressed by the treatment, but may not remain so. Interestingly, three exons showed the opposite response of splicing activation after depolarization (group 3). These results indicate a range of responses and regulatory mechanisms for exons affected by depolarization. In all, about 60% of the CaRRE-containing exons that were tested showed regulation by depolarization (Figure 7C and Table 1). As a control, we selected 17 alternative exons that do not contain any of the degenerate CaRRE motifs and tested their response to depolarization. As expected, the splicing of these exons was unaffected by depolarization. The one exception was Adcyap1r1 exon 14, which is discussed below (Figure 7A and 7B, group 2). Overall, we find that the presence of CaRRE motifs is highly predictive of depolarization-induced splicing regulation.

**Table 1:**
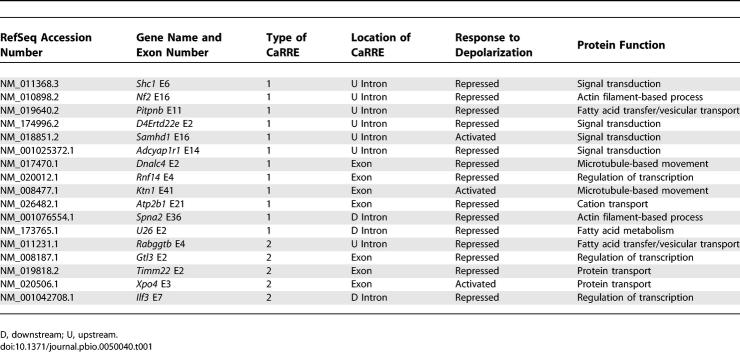
Summary of RT-PCR Analysis on CaRRE-Containing Exons

**Figure 8:**
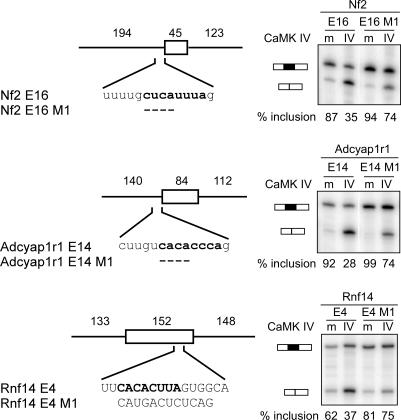
Mutagenesis Analysis Confirms the Activity of CaRRE 1 in the Regulated Exons Left: exons and the flanking introns were cloned from the mouse genomic DNA into pDup4–1. The length (in nucleotides) of each region is indicated above the diagram. The replaced and deleted nucleotides (dashed bar) in each mutant are shown below the wild-type sequence. Right: primer extension assay of the mutants indicated above.

To confirm that the predicted CaRREs were functioning in these regulated exons, we cloned 11 of these exons into the Dup4–1 splicing reporter and examined their splicing in HEK 293T cells. Six of these exons showed either complete inclusion or exclusion in the HEK 293T cells after transient expression (unpublished data ). Five exons showed partial inclusion. Of these, three exons showed CaMK IV–dependent repression in HEK 293T cells (Figure 8). For these exons, we tested the effect of CaRRE mutation on the CaMK IV–induced repression of these exons. Nf2 exon 16 contains a CaRRE 1 in the 3′ splice site whose deletion greatly reduces the CaMK IV effect (Figure 8). Rnf14 exon 4 contains an exonic CaRRE 1 whose replacement with the neutral IgM sequence again significantly reduces the CaMK IV repression. For Adcyap1r1 exon 14, no CaRRE was found in our motif search, but the exon was seen to be repressed by depolarization in differentiated P19 cells. Interestingly, a CACAYNNA sequence very similar to CaRRE 1 is seen in the 3′ splice site of this exon. Deletion of the CACA nucleotides of this element again significantly weakened the CaMK IV response. Thus a CaRRE 1–like element presumably also mediates the repression of this exon. These results confirm that the type 1 CaRREs in these exons are at least partially mediating their regulation. Unfortunately, none of the CaRRE 2–containing exons tested was spliced in the Dup construct in HEK 293T cells, and we were not able to confirm the role of this element for the computationally identified exons. Because the CaRRE 2 sequences were clearly functional in the CaMK IV repression assay (Figure 5B), behaving very similarly to CaRRE 1, CaRRE 2 is also likely to play a role in this response. However, its correlation with the group of depolarization-dependent exons is not as clear as CaRRE 1.

## Discussion

### Two Families of Regulatory Elements Mediate Depolarization-Induced Exon Repression

NMDAR1 E21 encodes the C1 peptide cassette that modulates a variety of activities of NMDARs. In this report, we show that the splicing of NMDAR1 E21 is repressed by depolarization in neurons. This repression requires CaMK IV activity and is reversible, suggesting that E21 splicing is finely tuned by dynamic inputs from the environment. In mutagenesis analyses, we identify two types of CaRRE motifs required for repression of NR1 E21 and demonstrate that either of these CaRRE motifs can be sufficient to confer CaMK IV–dependent repression on a heterologous exon. In bioinformatic searches, we find that these two CaRRE motifs define groups of co-regulated alternative exons. Thus, these regulatory elements found in NR1 E21 are controlling an ensemble of exons whose splicing is modulated in excitable cells.

Many known splicing regulatory motifs are short and degenerate, and we also observed these features in CaRRE 1 and CaRRE 2 [28,29]. In single nucleotide mutagenesis experiments, most base changes reduced activity, but some had only moderate effects. We also found that six CaRRE 1 and six CaRRE 2 mutations resulted in an improvement in silencing activity. It is likely that certain combinations of these mutations will also yield active elements. For CaRRE 1, the eight-nucleotide motif identified in E21 is slightly different from the 11-nucleotide motif located in the STREX 3′ splice site, but these two CaRRE 1 motifs share a CACAYN_1–4_A core sequence. The definition of these CaRRE motifs as a family of active elements allowed the identification of a group of co-regulated exons by database search. Of 27 exons carrying these motifs, 16 were confirmed as responsive by depolarization in P19 cells. In contrast, only one out of 17 exons not carrying a CaRRE motif showed repression after depolarization. The defined CaRRE motifs are thus highly predictive of exons regulated in this manner.

Notably, Adcyap1r1 exon 14 was the only exon tested that did not contain one of the eight-nucleotide CaRRE sequences used in the search, but was repressed after depolarization. Closer inspection identified a CACAYNNA sequence upstream of the AG dinucleotide in the 3′ splice site, and mutation of this element showed that it was important for the repression. Thus, there are clearly additional CaRRE 1 variants that we have not yet defined, and many additional co-regulated exons.

In addition to the CaRRE 1 motif, we identified a novel functional element GUGGUAGA in E21 that was also able to mediate the CaMK IV–induced repression. As with CaRRE1, this element has some intrinsic silencing activity even without CaMK IV, but does not appear to be similar to other identified exonic splicing silencers [29,30]. This new CaRRE type 2 motif behaved very much like the CaRRE type 1 motif, and could mediate splicing repression both in the E21 exon and when placed in the heterologous Dup175 exon. However, although the CaRRE 1 is enriched in alternative exons over constitutive exons, the CaRRE 2 motif frequency was similar in both types of exons. Because the presence of a CaRRE 2 was predictive for co-regulated exons, the lack of enrichment in the alternative exon set was puzzling and indicates the need to better define the family of functional type 2 CaRREs. There may be elements related to some portion of CaRRE type 2 that function in constitutive splicing and need to be distinguished from the repressor element itself. For example, one of the CaRRE2 variants included in the search contains a potential A1 binding site UAGG (Figure 5B, and see below). Given that the CaRRE 2 sequence was both essential for E21 repression and sufficient to induce repression on its own, it is clear that these variants of the CaRRE 2 motif play an important role in the response.

Recently, two exonic UAGG elements and one downstream intronic G tract were identified as important for the splicing repression of this exon [20]. To assess these elements relative to CaRRE 1 and CaRRE 2, we made mutations in them and tested their effect on inducible repression (M2, M4, and M5; Figure 3B). Consistent with the previous report, mutation of these silencers increased the splicing of E21. The different strength of splicing response that we observe may result from the different vector and cell systems used. Mutation of the UAGG motifs also reduced the repression by CaMK IV. However, the mutation of CaRRE 1 and CaRRE 2 motifs had much larger effects in the scanning mutagenesis. We also did not observe a correlation between the presence of UAGG and G tract motifs and the response to the depolarization in our other confirmed regulated exons. Although these other silencers may be direct targets of CaMK IV, it is possible that they serve to facilitate the CaMK IV repression by the CaRREs by weakening the overall splicing of E21. The cooperation of a CaRRE with an auxiliary silencer element was observed in the BK channel STREX exon [23]. This may also be what is seen in comparing the CaRRE1s from E21 and E5. Both elements mediate CaMK IV–inducible repression, but the element from E21, which contains a UAGG, is stronger (Figure 4C). Similarly, one of the CaRRE2 mutations that improved its activity created a UAGG. It will be interesting to look at the co-occurrence frequency of these splicing silencers with each CaRRE in regulated exons.

Alternative splicing events are generally regulated by complex interactions between multiple *cis*-acting pre-mRNA elements and *trans*-acting protein factors. Thus, the responsiveness of a CaRRE will depend on the sequence context of the element and the proteins expressed in each cell. In addition, most RNA elements can mediate either splicing repression or activation depending on their location and surrounding elements [31]. This may also be true for CaRRE 1, because the CaRRE1-containing exons in *Samhd1, Ktn1,* and *Xpo1* all increased in splicing with depolarization. Moreover, an exon that is responsive to depolarization in one tissue may not be in another, due to different protein expression profiles. Our finding that 60% of the CaRRE-containing exons were regulated by depolarization in the P19 cells is likely an underestimate of the percentage that is actually responsive, if one were to test other cells.

### The Role of CaRRE-Mediated Splicing Regulation in Cell Physiology

One determinant of synaptic activity is the number of neurotransmitter receptors present at the synapse [2,32]. This is frequently regulated by the protein trafficking systems of ER retention and export, and the transfer of proteins from vesicles to the plasma membrane. For NMDARs, these processes are affected by the splicing of NMDAR1 exons 21 and 22. Here we show that membrane depolarization in P19 cells represses the splicing of the NR1 E21, whereas the ratio of E22a to E22b remains the same. NR1 E21 encodes the C1 protein cassette containing an ER retention signal that ensures assembly of the NR1 subunits with the NR2 subunits in the ER [2]. The presence of C1 cassette changes the surface delivery rate of the assembled receptor. By comparing cortical cultures treated with either tetrodotoxin or bicuculline, the choice of E22a or E22b splicing was shown to be altered by neuronal activity [9]. This splicing of E22 also affected the rate of ER export, but the splicing of E21 was not seen to change with these treatments in these cells. Thus, in a variety of systems, alternative splicing appears to be a key mechanism for controlling delivery of NMDARs to the synapse and, presumably, for the regulation of cellular excitation and homeostasis.

The presence of a CaRRE is predictive of exons that change upon depolarization, and we used it to define a set of co-regulated exons. The broader identification of these regulated splicing events will be important in understanding the biological impact of depolarization on neurons. The functions of the genes so far identified are listed in Table 1, and affect a limited number of cell processes, including calcium homeostasis, intracellular signaling, and vesicular transport. Notably, depolarization regulates the splicing of exon 21 in the Atp2b1 transcript. Atp2b1 is a calcium ion pump that mediates the transport of calcium ions out of the cell [33]. Exon 21 of Atp2b1 encodes a calmodulin binding inhibitory domain. Repression of the inclusion of this domain will reduce the autoinhibition of Atp2b1 and hence facilitate the removal of calcium ions from the cell. This regulation of calcium homeostasis fits well with role of depolarization in inducing calcium influx through L-type calcium channels [34]. NMDAR1 and the BK channel are also proteins whose splicing alters ion movement across the membrane. The STREX exon affects the calcium and voltage sensitivity of the BK channels as well as its modulation by PKA [35], and alterations in its splicing will be affected by calcium homeostasis.

Other processes affected by depolarization-mediated splicing are protein and vesicular transport. *Pitpnb, Rabggtb, Dnalc4,* and *Ktn1* all affect vesicular transport and vesicle movement on microtubules, and *Xpo4* and *Timm22* affect the trafficking of proteins to different cell compartments. Depolarization also frequently regulates splicing in genes encoding signaling and scaffold proteins *(Shc1, D4Ertd22e, Samhd1, Adcyap1r1, Nf2,* and *Spna2).* We analyzed the functions of the 248 CaRRE-containing alternative exons using their associated SwissProt keywords (unpublished data). When compared to the parental dataset of 2,461 alternative exons, the CaRRE-containing exons showed enrichments of genes associated with the keywords *receptor, ion channel, calcium, kinase, protein phosphatase,* and *endocytosis*—all processes likely to affect the regulation of the synaptic activity. However, the confidence level of the enriched functional categories observed in this statistical gene ontology analysis was not high. By identifying more CaRRE-containing alternative exons, we hope to improve this analysis.

In the brain, neurons constantly receive signals from other cells and adjust their activity according to the incoming stimuli. In this study, we looked at the splicing response to chronic depolarization and observed several new interesting features of this regulation. We find that the extent of the splicing repression is dependent on the KCl concentration, and that splicing repression is reversible after the removal of the KCl from the medium. Thus, the splicing repression is affected by the strength of the external stimuli, and can respond to the dynamic nature of the cellular environment. We also find that different exons respond to the same treatment with different dynamics. Some exons maintain their repressed level throughout the depolarization time course, whereas other exons adapt to the stimulus and start to recover while still in elevated KCl concentrations. Finally, we find that depolarization not only represses, but also activates, splicing of some exons in neuronal cells. All these properties will allow the fine tuning of individual splicing patterns in excitable cells.

The identification of the proteins that bind to the CaRRE motifs within E21 will be needed to understand the mechanism of the inducible splicing regulation. The characterization of the CaRREs in this study will allow the use of biochemical approaches to address this question. Other important questions include what additional molecules are required in the CaMK IV pathway, whether other signaling pathways activated by depolarization also participate in splicing regulation, and how these signaling pathways affect the splicing apparatus. Finally, these responses can look quite different at the single-cell level from the changes observed in bulk culture. Thus, single-cell assays of this splicing regulation are needed. Experiments that address some of these questions will lead to a better understanding of the role of splicing regulation in neuronal cell biology.

## Materials and Methods

### Cell culture.

HEK 293T cells were grown in DMEM with 10% fetal bovine serum (FBS), and P19 cells were grown in αMEM with 10% FBS. To induce differentiation of P19 cells, the cells were transferred to induction medium containing αMEM with 5% FBS and 0.5 μM all-trans retinoic acid (Sigma-Aldrich R-2625; Sigma-Aldrich, St. Louis, Missouri, United States) in bacteriological Petri dishes to promote cell aggregation. After 2 d, cell aggregates were transferred to fresh induction medium and cultured for another 2 d. The cell aggregates were then trypsinized and 5 × 10^6^ cells were plated per 100-mm tissue culture plate in αMEM with 10% FBS. Two days after plating, 30 μM arabinocytidine (ARAC, Sigma-Aldrich C1768) was added to the media to inhibit proliferation of non-neuronal (glial-like) cells.

For depolarization, day 10 cultures of differentiated P19 cells were shifted to medium containing 20 to 50 mM KCl with or without CaMK IV inhibitor KN93 (30 μM) or the inactive analog KN92 (30 μM). For the recovery experiments, day 9 cultures were used for depolarization and after 18 or 24 h, the KCl-containing medium was replaced with fresh culture medium, and the cells were incubated for another 24 h.

### RT-PCR assay.

One microgram of the cytoplasmic RNA was reverse transcribed with a mixture of ten specific primers against the target exons (0.1 nmole each), and one tenth of the reaction was then amplified in 22–25 cycles of PCR with exon-specific primers, one of which was ^32^P-labeled. The PCR products were resolved on 8% polyacrylamide/8 M urea denaturing gels. The gel was dried, exposed, and scanned in a PhosphorImager (Fuji Medical Systems, Roselle, Illinois, United States).

### Plasmid construction and mutagenesis.

Exons and partial flanking introns of the regulated exons were amplified using Pfu DNA polymerase from mouse genomic DNA and subcloned between the ApaI and BglII sites of pDUP4–1. In the single nucleotide scanning experiments, the ApaI site was mutated to XhoI site to facilitate cloning. Mutations were made by PCR using Pfu DNA polymerase. The sequences and mutations were confirmed by DNA sequencing.

### Transfection and primer extension assay of minigene splicing reporters.

HEK 293T cells were grown in six-well plates to about 60% confluence and transfected using Superfect (Qiagen, Valencia, California, United States) with 1 μg of the minigene reporter and 2 μg of the CaMK IV-dCT or CaMK IV-dCTK75E expression construct. Cytoplasmic RNA was purified after 24 h and one third was used in a reverse transcription reaction using Superscript II (Invitrogen, Carlsbad, California, United States) with ^32^P-labeled DNA oligo DUP3 (AACAGCATCAGGAGTGGACAGATCC). Half of the extension product was then mixed with an equal volume of loading buffer, denatured, and then resolved on 8% polyacrylamide/8 M urea denaturing gels. The gel was dried, exposed, and then scanned in a PhosphorImager.

### Generation of datasets and computational analysis.

We detected alternative splice forms in mouse by mapping mRNA and ESTs onto genomic sequences as previously described [36,37] using the following data: (1) UniGene EST data [38] from January 2006 for mouse (ftp://ftp.ncbi.nih.gov/repository/UniGene/Mus_musculus) and (2) mouse mm7 genome assembly (http://hgdownload.cse.ucsc.edu/goldenPath/mm7/chromosomes). Internal exons were identified as genomic regions flanked by two splices, and all exon boundaries were confirmed by checking consensus splice site motifs. We identified a total of 5,497 cassette exons in 3,593 mouse genes. Of these, 2,594 cassette exons in 1,929 mouse genes had multiple ESTs, supporting both the exon-inclusion and exon-skipped forms, and were used in our subsequent analysis. We also extracted a random set of 10,000 constitutive exons as our control.

The CaRRE consensus sequences were used to search the exons and a 50-nucleotide region of the upstream and downstream introns in our datasets. This was done with scripts written in Perl. For the frequency analysis, the occurrences of the CaRRE 1 or 2 consensus sequences were compared to the occurrences of random octamers. The *p*-values were calculated using the Fisher's exact test.

## Supporting Information

Figure S1 Depolarization Induces Splicing Repression of NMDAR1 Exon 21 to Exon 22b in Differentiated P19 Cells (A) Depolarization represses the splicing of E21 to E22b in differentiated P19 cells. Left panel: denaturing gel electrophoresis of the NR1–3 and NR1–4 RT-PCR products from day 10 differentiated P19 cells depolarized with 50 mM KCl for 0, 6, 12, 18, or 24 h (lanes 1–5), from day 11 untreated cells (lane 6), and from day 10 cells depolarized with 20, 30, or 40 mM KCl for 24 h. Right panel: percentage of mRNA containing E21 after depolarization ((NR1–3/(NR1–3 + NR1–4))*100). Bars indicate mean ± standard deviation (s.d.), *n* = 3.
(B) Depolarization induced repression is dependent on CaMK. Left panel: RT-PCR assay of NR1–3 and NR1–4 mRNA from differentiated P19 cells treated with KCl, KCl plus KN93, or KCl plus KN92 for 12 h. Right panel: graph of E21 inclusion as shown in the left panel.
(C) Splicing repression is reversible after removal of the stimulus. Left: RT-PCR assay of NR1–3 and NR1–4 mRNA isolated from cells depolarized with 50 mM KCl for 18 or 24 h and 24 h after the salt washout. Right: graph of E21 inclusion as shown in the left panel.
(1.3 MB TIF)

Table S1 The Positions and Sequences of the CaRREs in the Regulated Exons (18 KB XLS)

### Accession Numbers

The GenBank (http://www.ncbi.nlm.nih.gov/Genbank/) RefSeq accession numbers for the genes discussed in this paper are as follows: *Adcyap1r1* (NM_001025372.1), *Atp2b1* (NM_026482.1), *D4Ertd22e* (NM_174996.2), *Dnalc4* (NM_017470.1), *Gtl3* (NM_008187.1), *Ilf3* (NM_001042708.1), *Ktn1* (NM_008477.1), *Nf2* (NM_010898.2), *Pitpnb* (NM_019640.2), *Rabggtb* (NM_011231.1), *Rnf14* (NM_020012.1), *Samhd1* (NM_018851.2), *Shc1* (NM_011368.3), *Spna2* (NM_001076554.1), *Timm22* (NM_019818.2), *U26* (NM_173765.1), and *Xpo4* (NM_020506.1).

## Abbreviations

CaMK: Ca++/calmodulin-dependent protein kinase
CaRRE: Ca^++^/calmodulin-dependent protein kinase IV–responsive RNA element
ER: endoplasmic reticulum
EST: expressed sequence tag
hnRNP: heterogeneous nuclear ribonucleoprotein
NMDAR: NMDA receptor
SR: serine/arginine-rich

## Note Added in Proof

Ping An and Paula Grabowski have completed a similar analysis of NR1 exon 21 (PLoS Biol this issue). They confirm a role for A1 sites in mediating the effects of depolarization on E21 splicing and demonstrate that this regulation occurs in primary cortical neurons.

An P, Grabowski PJ (2007) Exon silencing by UAGG motifs in response to neuronal excitation. PLoS Biol 5: e36. doi:10.1371/journal.pbio.0050036
